# Daily surgery caseload prediction: towards improving operating theatre efficiency

**DOI:** 10.1186/s12911-022-01893-8

**Published:** 2022-06-07

**Authors:** Hamed Hassanzadeh, Justin Boyle, Sankalp Khanna, Barbara Biki, Faraz Syed

**Affiliations:** 1 The Australian e-Health Research Centre, CSIRO, Brisbane, QLD Australia; 2 Fiona Stanley and Fremantle Hospital, WA Health, Perth, Australia; 3 Level 7, Surgical, Treatment and Rehabilitation Service–STARS, 296 Herston Road, Herston, QLD Australia

**Keywords:** Demand forecasting, Operating theatre efficiency, Critical care

## Abstract

**Background:**

In many hospitals, operating theatres are not used to their full potential due to the dynamic nature of demand and the complexity of theatre scheduling. Theatre inefficiencies may lead to access block and delays in treating patients requiring critical care. This study aims to employ operating theatre data to provide decision support for improved theatre management.

**Method:**

Historical observations are used to predict long-term daily surgery caseload in various levels of granularity, from emergency versus elective surgeries to clinical specialty-level demands. A statistical modelling and a machine learning-based approach are developed to estimate daily surgery demand. The statistical model predicts daily demands based on historical observations through weekly rolling windows and calendar variables. The machine learning approach, based on regression algorithms, learns from a combination of temporal and sequential features. A de-identified data extract of elective and emergency surgeries at a major 783-bed metropolitan hospital over four years was used. The first three years of data were used as historical observations for training the models. The models were then evaluated on the final year of data.

**Results:**

Daily counts of overall surgery at a hospital-level could be predicted with approximately 90% accuracy, though smaller subgroups of daily demands by medical specialty are less predictable. Predictions were generated on a daily basis a year in advance with consistent predictive performance across the forecast horizon.

**Conclusion:**

Predicting operating theatre demand is a viable component in theatre management, enabling hospitals to provide services as efficiently and effectively as possible to obtain the best health outcomes. Due to its consistent predictive performance over various forecasting ranges, this approach can inform both short-term staffing choices as well as long-term strategic planning.

**Supplementary Information:**

The online version contains supplementary material available at 10.1186/s12911-022-01893-8.

## Background

Operating theatres are one of the costliest components in hospital care. Studies have quantified theatre inefficiency costs (calculated by multiplying the time wasted with staff capacity costs and opportunity costs) at ~ $50/min or approximately $2000/day/theatre [1]. However, there are many reasons contributing to theatre inefficiency including hospital-wide factors such as availability of ward beds, transfer of patients and poor pre-operative preparations, as well as doctor-related factors such as the unavailability of surgeons, anaesthetists and nurses. Improving theatre efficiency should therefore be at the forefront of efforts to improve health service efficiency. Various approaches have been explored towards optimising theatre efficiency, from predicting surgery duration and operating theatre scheduling optimisation [2, 3] to predicting surgery demand [4, 5], which can help improve the utilisation of theatres, assist with staffing, and reduce patient waiting times.

The literature in predicting patient demand mostly consists of approaches to forecast overall daily demand from a hospital-level perspective using statistical modelling and time series analysis techniques [6–13]. There are few studies that attempted predictions at a finer granularity, and more specifically, for daily surgical caseloads [4, 5]. In general, the majority of related approaches used seasonal auto-regressive integrated moving average (ARIMA) techniques that are more reliable for short-term forecasts. In order to extend the forecasting horizon, recent approaches adopt machine learning models and combine these with time series techniques [14–18]. However, these approaches mainly focused on predicting daily demands of Emergency Departments (ED) or outpatient clinics. There is a gap in the literature for a more focused approach as a means for improving theatre management and strategy planning by providing realistic estimations of theatre demand across both short- and long-term horizons.

In this study we developed statistical and machine learning models to predict daily surgery caseload to an extent of up to one year ahead. Factors associated with theatre demand were investigated and a number of candidate variables were selected to train the predictive models, such as day-of-week, time of year, and local factors such as public holidays. When testing predictive accuracy, a recommended approach is to divide historical data into a training dataset and testing dataset and measure the error from the model on data not used in building the model. Unlike some industrial applications, daily counts of patient arrivals to theatre are not independent and it is not appropriate to select a validation period using random periods within the entire dataset (using for example tenfold cross validation). The data instead has some dependence based on time (chronologically ordered) and thus the validation period for time series modelling is chosen as the last complete year (to account for seasonal differences) in the dataset. It was desirable to also determine the effect of using different lengths of data for training the model: e.g., using the most recent year versus all available data. It is believed that predicting theatre demand can help improve the ability to optimise theatre templates and case selection beyond current ad-hoc approaches and ensure that more patients are cared for in a planned fashion within a more efficient utilisation of theatre time. A new set of features to encode historical surgery demand patterns was developed that were used as input to several machine learning models for predicting daily surgery caseloads in different urgency levels. In addition, a fast and practical approach based on statistical modelling for daily surgery caseload forecasting in different urgency and specialty levels was also presented. A thorough comparison of all these models and their feasibility for surgery demand prediction was assessed. While the literature on surgery demand forecasting mostly focuses on short-term predictions for up to 6 months in advance, in our study we aimed for a wide forecasting horizon up to one year including weekends and public holidays allowing accuracy to be measured across summer and winter months. Our approach was able to reliably predict the daily surgery caseload with approximately 10% Mean Absolute Percentage Error (MAPE) across a one-year forecast horizon while a 16% error was evident in the literature across a shorter 6-month forecast horizon.

## Related work

This section provides a more detailed review of prior art in forecasting healthcare demand in various settings. Jilani et al. [10] presented a Fuzzy Time Series (FTS) approach for forecasting daily emergency department demand. They developed separate FTS models for each weekday to capture variations in ED attendances. They compared FTS with ARIMA and neural network (NN) models that were tested over a dataset comprising admissions from four EDs. The FTS approach was able to predict ED demand with almost half the error of ARIMA and NN models. Compared to their approach, our model provides more fine-grained forecasts of daily emergency and elective surgery caseloads. In addition, our approach provides demand forecasts up to one year ahead compared to the 4-month period of that study which may be subject to biases associated with the time of year.

Jones et al. [19] evaluated several models, including seasonal ARIMA, time series regression, exponential smoothing, and artificial neural networks for forecasting ED demand on data from three facilities. Their forecast horizon was from 1 day to a maximum of 30 days, which is a considerably shorter evaluation period compared to our present study. The models exhibited mixed results with MAPE of 9 to 14%.

Calegari et al. [8] employed a number of time series models to forecast demand of patients based on the level of their urgency (five triage categories). The forecasting horizon in their approach spanned from 1 to 30 days and the best performing model showed MAPE ranging between 2 and 11% for total patients. They also tried to incorporate climate factors into their models, which they concluded did not improve the performance of the models. Similarly, in our study we attempted to provide daily surgery caseload forecasting in different granularities, covering elective and emergency streams and the top-10 most frequent specialties, while considering a longer forecast horizon for evaluation.

Luo et al. [20] applied seasonal ARIMA and single exponential smoothing models and a combination of them to forecast daily outpatient visits for one week ahead. Using 43 weeks of observation data, their combinatorial model performed relatively better than the individual models and showed MAPE values of between 11 and 13% for forecasting demand for endocrinology and respiratory departments.

While the majority of the approaches for demand forecasting in the literature are based on time series models, there have been attempts to employ machine learning algorithms for this purpose. Wang et al. developed a hybrid approach by using Support Vector Regression and the firefly algorithm, an optimization algorithm, for forecasting diarrhoeal outpatient visits [21]. The input to their model included daily temperature, relative humidity and rainfall, as well as historical daily outpatient visits in Shanghai for six years, the last year reserved for testing the model. They divided the patients into two groups of children and adults, for which their model performed in the best setting with 7% and 11% MAPE, respectively. In our study, we also developed and validated several machine learning models for daily surgery caseload forecasting for a long forecast horizon of one year. There remains a gap in the literature for such an experiment on the application of these models for surgery demand prediction that can inform operating theatre management team as well as hospital executives to better plan for maximising operating theatre utilisation.

There is scant published literature that specifically focuses on forecasting surgery demand. Tiwari et al. presented an approach for predicting daily elective surgery volume for up to 14 days in advance [4]. They collected daily operating theatre schedules for a period of 8 months, and for any given day in the forecasting horizon, data from the prior 30 days was used to predict the case volume of the day based on linear regression modelling. In 80% of cases their model predicted with ± 7 cases deviation for a week ahead. Eggman et al. validated the generalizability of the days-out linear regression modelling in Tiwari et al. while exploring the significance of further independent factors in predicting daily surgical volume [22]. They tested the model over data from two hospital-based operating room campuses at an academic medical center and showed that the predicted volumes were within 7 cases (error) for 81% of days and 69% of days at the two sites. Finally, Zinouri et al. [5] applied seasonal ARIMA to provide short-term forecasts of daily surgical demand. Their model showed MAPE of 7% for forecasting demand for one week ahead and 16% for 6 months ahead.

Overall, the literature shows that providing accurate forecasts of surgery caseload in the weeks and months ahead can help daily operational planning of staff and resources as well as providing insights for longer strategic planning of critical care services in operating theatres. The identified gaps in the literature are around the absence of a model for daily surgery caseload forecasting that is capable of forecasting for a longer horizon and for several levels of granularity in terms of urgency of cases (elective or emergency) or medical specialty, as well as a lack of consideration of all working and non-working days (public holidays) in the modelling. As a result, our study presents models that can reliably and comprehensively forecast daily surgery demand across both short- and long-term horizons for a variety of surgery cases throughout a year.

## Methods

**Fig. 1:**
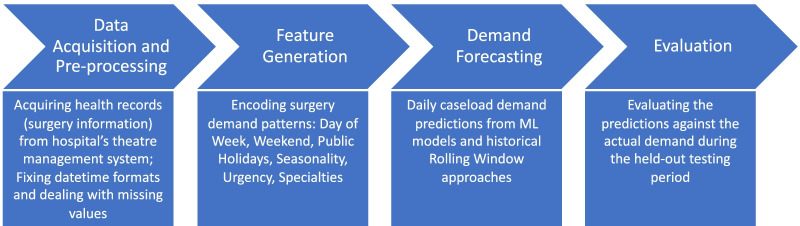
Flow diagram of daily surgery caseload prediction

Figure 1 shows an overview of the process from a data acquisition step to validating the models for the daily surgery caseload prediction task. The rest of this section provide more details on each step in this process.

### Data

The data for this study was sourced from an administrative database containing information about elective and emergency surgeries. A data extract that included de-identified records of patients who had undergone surgery in one of Australia’s major metropolitan hospitals was collated.^1^ All surgery episode records at the session level, operation level and procedure level for a period of 4 years were collected, from 1 January 2016 to 31 December 2019. The data consists of 99,732 surgeries on 63,697 unique patients. The pre-processing step involved fixing inconsistencies in date/time formats, fixing missing values in essential timestamps (filled with adjacent timestamps), and fixing overlapping operations.

**Fig. 2:**
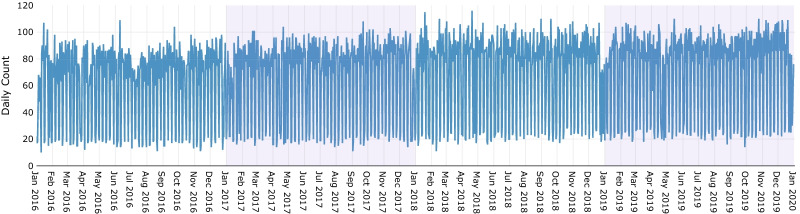
Count of daily surgery (emergency and elective) during the study period

Figure 2 shows the number of surgeries performed per day during the study period. Large differences in the number of surgeries per day typically relate to more operations performed during weekdays as opposed to weekends and public holidays.

**Fig. 3:**
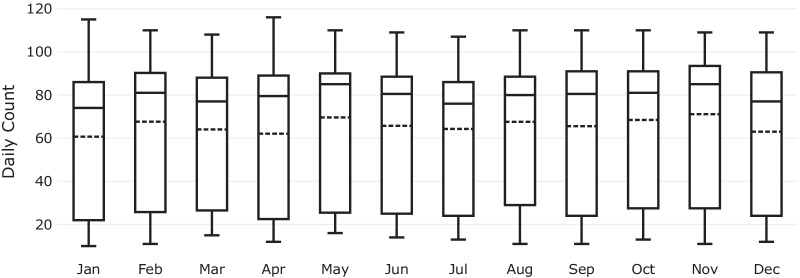
Daily all surgery count (emergency and elective) per month during the study period

Figure 3 shows the number of surgeries per month during the study period.^2^ It can be observed that there were generally less surgeries performed in January, April, and December months with 61, 62, and 63 mean daily counts, respectively, indicating fewer scheduled elective operations due to major public holidays. November had the highest mean daily count of 71, followed by May, August, and October with 70, 68, and 68 mean daily counts, respectively.

**Fig. 4:**
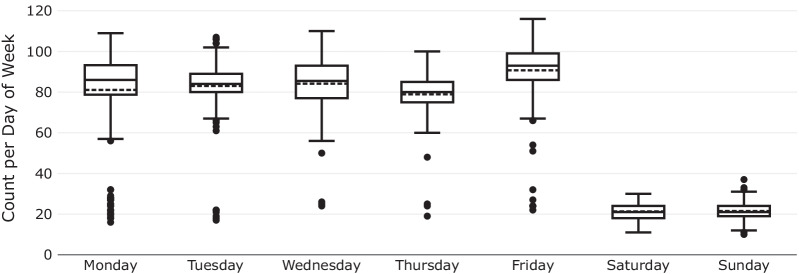
Count of all surgery (emergency and elective) per day of week during the study period

Figure 4 shows the volume of surgeries per day of week during the study period. The average number of operations on Fridays (93) was relatively higher than the rest of the days. The remaining working days had mean counts between 79 to 84 operations. There were only 21 operations on average on Saturdays and Sundays.

In order to develop and evaluate the predictive models, the data was divided into training and testing periods:

- Training period: 1 January 2016 to 31 December 2018 (3 years)
- Testing period: 1 January 2019 to 31 December 2019 (1 year).

Data was divided based on type of surgery (i.e., emergency or elective) and medical specialty. Daily arrivals to operating theatre according to each of these specifications were calculated and formed the input to predictive models.

### Predictive models

Two different predictive models were developed to predict operating theatre demand on a daily basis: a statistical modelling approach and a machine learning based approach. These approaches are described in the following subsections.

#### Rolling window on historic observations

This approach employs information that is carried in historic observations in relation to time-related characteristics [23]. More specifically, to predict daily patient admissions in a point of time in the future, different temporal aspects such as seasonality, day of week, and public holidays were considered and historic observations were queried according to these aspects. Different rolling windows (time-frames) are considered in this approach when collecting historic observations. This approach has the following two modules:

- Observation Collection Module: Collects historic observations related to a target date.
- Prediction Module: Forecasts the demand for the target date based on the collected observations.

The observation collection module is described in detail in Algorithm 1. Note that, Algorithm 1 describes the module in a “validation” setting and the days of interest (DOIs) were selected from the testing period (as described in section “Data”) to evaluate the model. A DOI can be any date in future in an “application” setting. *DOI*_*year*_ in Algorithm 1 refers to the “year” component of the given date (e.g., “2020” in DOI = 2020/03/12). For any DOI, the algorithm collects matching historic observations in a weekly window preceding the DOI in the current year and surrounding weekly windows in preceding years. Retrieving historic observations in a yearly manner can be customised by the parameter τ (e.g., if τ = 2 then the related observations from the preceding two years of the given target date would be collected), and the weekly rolling windows can be customised with parameter θ (e.g., if θ = 2 then observations from two weeks before and two weeks after the corresponding date in a preceding year would be collected).
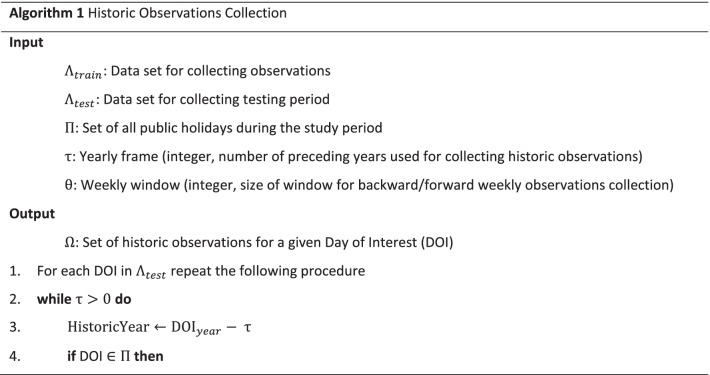


In Algorithm 1, a corresponding day refers to the same day of week and week of year as the DOI, but in a preceding year. So, the date of the corresponding day may not be exactly the same (in terms of month and day) as the DOI.

**Fig. 5:**
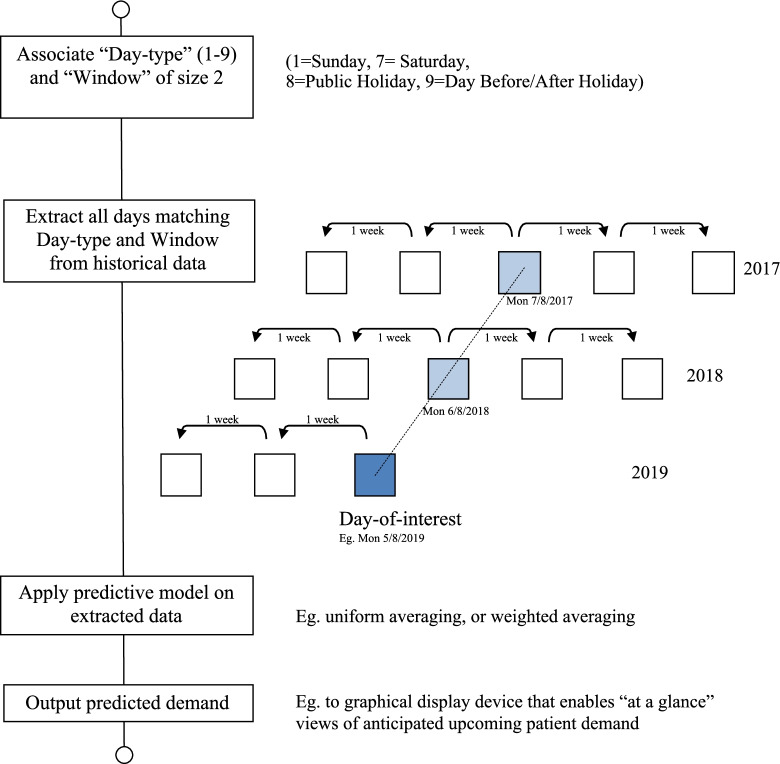
The Rolling Window approach logic

The prediction module of this model takes the set of retrieved observations (i.e., the output of the observation collection module) and predicts the demand for a DOI based on a mathematical function. A range of functions can be applied on the set of collected observations, such as, weighted mean, maximum, minimum, etc. In this study, a uniform weighted mean was imposed on the collected observations (i.e., similar weights were assigned to the observations from all preceding years). Figure 5 shows the logic behind the Rolling Window approach using values of τ = 2 and θ = 2 to illustrate the method.

#### Regression models

As described earlier, the prediction task in this study is to forecast elective, emergency, and overall daily surgery demand for a given calendar day. For training the regression models and in order to represent characteristics of each calendar year, a set of temporal features were extracted from the data. To encode the sequential order of the observations, a relative daily index was assigned to each day that started from the first day in the specified training period and continued until the end of testing period. Day of week was another feature encoded in the feature vector. In addition to that, the effect of public holidays was also represented as an additional type of day.

To model the repeated patterns due to seasonality, we adopted the first order Fourier series [24]:

1 $${\text{Sin}} \left( {\frac{2\pi t}{m}} \right), {\text{Cos}} \left( {\frac{2\pi t}{m}} \right)$$

where, *t* refers to a day in our study period, and *m* is the seasonal period, which is 365 days in our model.

A number of regression models were employed in this study to investigate their effectiveness in predicting daily surgery demand: Linear Regression (normal, Poisson, and Negative Binomial families) [25], Decision Tree [26], Random Forest [27], Support Vector Regressor (SVR) [28], Bagging Regressor [29], Gradient Boosting Regressor [30], XGBoost [31], and an Ensemble Regressor. Our Ensemble Regressor was composed of a uniform weight voting algorithm with Random Forest, Bagging, and Gradient Boosting regression models as base predictive models.

### Experimental setup and evaluation measure

Data manipulations and the predictive models were implemented in the Python programming language version 3.9. The regression models were implemented using Python’s *Scikit-learn* and *Statsmodels* toolkits and the details of their hyper-parameters and the tuning strategy can be found in the Additional file 1: Appendix A: Regression Model Hyperparameters Tuning [32, 33]. The “seed” value for the stochastic algorithms was set to “1”.^3^

In validating predictive accuracy, we measured the error every day across our testing period between actual observations and predicted values generated from a model using training data only (i.e. maintaining a separate held out evaluation period), and followed established principles regarding the assessment of forecast accuracy [34].

If *Y*_*t*_ is the actual observation for time period *t* and *F*_*t*_ is the prediction for the same period, then the error is defined as *e*_*t*_ = *Y*_*t *_− *F*_*t*_. If there are *n* observations then the Mean Absolute Error (MAE–or Mean Absolute Deviation MAD), Mean Squared Error (MSE), and Root Mean Squared Error (RMSE) of predictions can be defined as:

2 $${\text{MAE}} = {\text{MAD}} = \frac{1}{n}\mathop \sum \limits_{t = 1}^{n} \left| {e_{t} } \right|$$

3 $${\text{MSE}} = \frac{1}{n}\mathop \sum \limits_{t = 1}^{n} e_{t}^{2}$$

4 $${\text{RMSE}} = \sqrt {\frac{{\mathop \sum \nolimits_{t = 1}^{n} e_{t}^{2} }}{n}}$$

In order to provide a scale-independent measure, Mean Absolute Percentage Error (MAPE) is reported in the manuscript. MAPE is based on the Percentage Error of forecasts (*PE*_*t*_), which is defined as:

5 $$PE_{t} = \left( {\frac{{Y_{t} - F_{t} }}{{Y_{t} }}} \right) \times 100$$

Using this relative error, MAPE can be calculated as:

6 $${\text{MAPE}} = \frac{1}{n}\mathop \sum \limits_{t = 1}^{n} \left| {PE_{t} } \right|$$

## Results

### Predicting daily surgery demand

Table 1 summarises the results (in terms of MAPE, the average of absolute percentage errors across the entire test period) of all forecast approaches using different lengths of training data applied to the prediction of emergency and overall surgery (overall surgery comprises emergency and elective surgery). The underlined values in Table 1 indicate the best performance in each column which assesses the effect of using different lengths of training data (i.e., 1-Year, 2-Year, or 3-Year). Boldfaced values refer to the overall best performance for the specified type of surgery (i.e., emergency, elective or overall surgeries). The weekly window size ($\uptheta$) for the Rolling Window approach in Table 1 was set to 2: 2 weeks before and 2 weeks after a given day of interest (for the full set of results with different weekly window sizes see Additional file 1: Table B.1 in Appendix B: Extended Results). Note that the reported predictive performance for elective surgery demand excludes weekends and public holidays caseload due to instances of no elective surgery undertaken on these day types which affects the calculation of MAPE.

**Table 1:** Daily surgery demand prediction results (MAPE)

	Emergency surgery			Elective surgery			Overall surgery		
	1-year %	2-year %	3-year %	1-year %	2-year %	3-year %	1-year %	2-year %	3-year %
Rolling window	11.79	12.05	13.66	13.26	12.61	12.97	**9.52**	9.83	11.31
Regression (Linear)	N/A	13.71	13.79	N/A	17.79	15.84	N/A	13.49	12.85
Regression (Poisson)	N/A	14.17	14.71	N/A	18.90	16.68	N/A	13.99	13.77
Regression (Negative binomial)	N/A	14.13	14.88	N/A	19.12	16.67	N/A	14.08	13.86
Decision tree	15.69	17.97	18.30	14.55	13.91	14.72	15.99	13.97	12.14
Random forest	11.73	11.50	11.63	16.41	**11.51**	12.99	10.44	9.86	9.88
SVR (Linear)	19.13	16.50	17.50	15.61	15.62	15.19	35.81	37.93	36.96
SVR (RBF)	22.35	19.37	17.86	13.60	13.65	13.78	74.68	57.40	48.15
SVR (Sigmoid)	20.49	51.91	50.95	15.19	18.94	21.02	60.37	50.05	51.49
SVR (Poly)	44.12	26.67	23.63	19.23	21.31	15.55	90.15	48.84	36.43
Bagging regressor	12.86	11.97	12.50	13.62	11.90	14.25	10.62	10.42	10.26
Gradient boosting regressor	13.25	11.69	**11.27**	11.63	16.52	14.00	10.74	11.21	10.61
XGBoost regressor	13.60	13.65	14.89	37.18	15.74	40.92	15.06	11.64	11.22
Ensemble regressor	12.36	11.46	11.61	12.99	12.69	13.30	10.33	9.97	9.82

The underlined values indicate the best performance in each column

Boldfaced values refer to the overall best performance

In general, the performance of the Rolling Window approach decreased when longer data histories were employed for predicting emergency and overall surgeries. For elective surgeries, using only one year of historic observations led to more error (i.e., 13.26%) while using two years of observations resulted in the least error (i.e., 12.61%).

The regression models demonstrated mixed benefits from adopting different lengths of training data. For example, the Linear, Poisson, and Negative Binomial Regression models were not able to achieve reasonable results using 1 year of historical data (MAPE values for these models were higher than 100%). As more historic observations were used in these models, improvements in predictive accuracy were observed for all three groups of emergency, elective, and overall surgeries. Apart from predicting the elective surgery demand relatively well, the SVR models generally showed poor performance using different length of historical data. The remaining approaches do not exhibit a consistent pattern in regards to the effect of different lengths of training data. The Gradient Boosting Regressor achieved the best performance for emergency surgeries with 11.27% MAPE and the Rolling Window achieved the best performance for predicting overall surgeries with 9.52% MAPE. For elective surgeries, the Random Forest model reached the lowest MAPE (i.e., 11.51%) using two years of training data (for the full set of results with different weekly window sizes see Additional file 1: Table B.2–Table B.4 in Appendix B: Extended Results). Significance testing based on pairwise two-tailed t-test comparisons with corrections for multiple testing using the Bonferroni correction [35] revealed that the differences in error between the Rolling Window and the best performing regression models were not statistically significant except for emergency surgery with 3-years of training data (where random forests, boosting and ensemble approaches were best) and for elective surgery with 1-year of training data (where the Rolling Window approach was best). Given that on balance the Rolling Window approach has either the least error or is statistically similar to the best approach, and that the model is more deterministic (i.e., there is no random number generator component) and its forecasts are more interpretable, the rest of the results presented in this section are based on the results of the Rolling Window model.

**Fig. 6:**
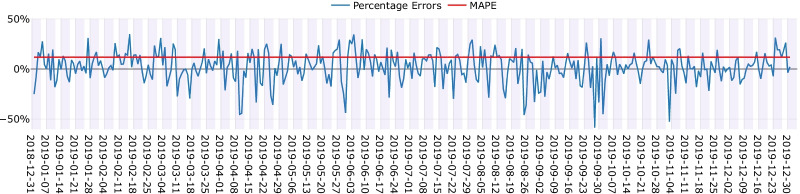
Daily prediction errors for emergency surgeries over the testing period using the Rolling Window approach (shaded areas indicate working days)

Figure 6 shows the daily percentage errors and MAPE of the Rolling Window approach for emergency surgeries (using the final year of historical observations and weekly windows of size 2) during the testing period. Shaded areas in Fig. 6 indicate Monday to Friday working days and the white spaces are Saturdays and Sundays. Following Eq. 5, negative Percentage Errors indicate that the predictions were higher than actual observations (over-estimations) while positive Percentage Errors refer to when the predictions were lower than the actual observations (under-estimations). The majority of the under-estimated days were weekends (i.e., days with high positive percentage errors) while the over-estimations mostly happened during working days (i.e., days with high negative percentage errors). The largest under-estimation by the system was on Saturday 16 February 2019 with + 35% error. There were 27 emergency surgeries on this day while historically there were only 12 to 24 surgeries, and the model’s prediction for this day was 18. The most considerable over-estimation happened on Saturday 28 September 2019 with more than − 50% error. The actual number of surgeries on this day was 13 while the model prediction was 21. The historical observations for this day suggested a range of 15 to 28 surgeries while on that particular Saturday in 2019 there were relatively fewer operations. Another interesting aspect that can be observed from Fig. 6 is that the Rolling Window approach showed consistent performance in predicting emergency surgery demand for a year ahead and its errors for the days in the beginning and end of the testing period did not vary considerably. This can be a critical aspect for hospitals when conducting long-term resource management based on the outputs of a predictive model.

One of the days with the least surgery during the testing period was Friday 26 April 2019, with 33 emergency cases. The model over-estimated the caseload for this day with − 35% error. This day immediately followed a public holiday (Thursday 25 April 2019) in addition to being after a long weekend from Friday 19 April to Monday 22 April. A lower demand on such a day was anticipated (due to potential higher recreational travel). However, the model’s poor prediction was due to the fact that such a series of public holidays was not evident historically (e.g., there was only one public holiday on 25 October 2018 without any preceding or succeeding holidays in a two-week window size). One of the days with highest emergency surgery demand (51 cases) during the testing period was Friday 14 June 2019. For such a unique day in terms of demand, but a normal weekday, the model under-estimated the caseload with 14% error (approximately 7 less surgeries than the real number).

**Fig. 7:**
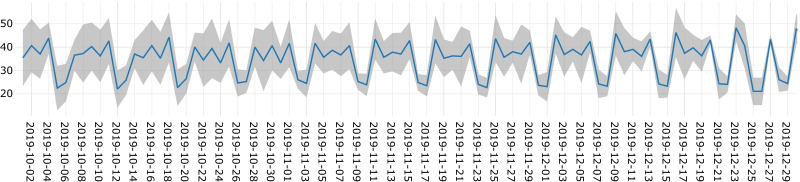
Daily predictions with 95% confidence intervals for emergency surgeries during the final three months of the testing period using the Rolling Window approach

Figure 7 shows the daily emergency surgery demand prediction and their 95% confidence intervals during the final three months of the testing period. It can be observed that the confidence intervals have a consistent range throughout the last three months of the predictions.

**Fig. 8:**
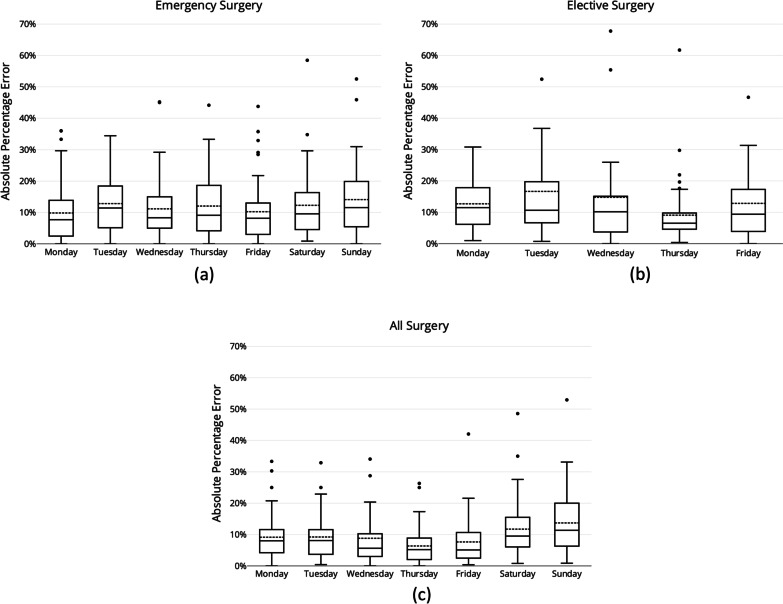
Error per day of week for the Rolling Window approach: **a** emergency surgeries, **b** elective surgeries, and **c** all surgeries

Figure 8 shows the absolute percentage errors of the rolling window approach by day of week for emergency surgeries (Fig. 8a), for elective surgeries (Fig. 8b), and for all surgeries (Fig. 8c). For both emergency and all surgeries, the Rolling Window approach showed higher predictive error over the weekend with 12% and 14% MAPE on Saturdays and Sundays respectively (note that weekends are excluded for elective cases following the planning processes for elective cases at the hospital). This approach predicted emergency surgeries on Monday and Fridays more accurately than other working days with 10% MAPE (Fig. 8a). The MAPE for predicting all surgeries across working days were relatively lower in the range of 6% to 9% (Fig. 8c). Elective surgery error was highest on Tuesdays with 17% average error (MAPE), followed by Wednesdays and Fridays with 15% average error (Fig. 8b). The average errors of the model for Thursdays were relatively lower with 9% MAPE. When compared to predicting daily emergency demand (as illustrated in Fig. 8a), generally the model produced slightly higher errors when predicting elective demand during working days than emergency demand.

**Fig. 9:**
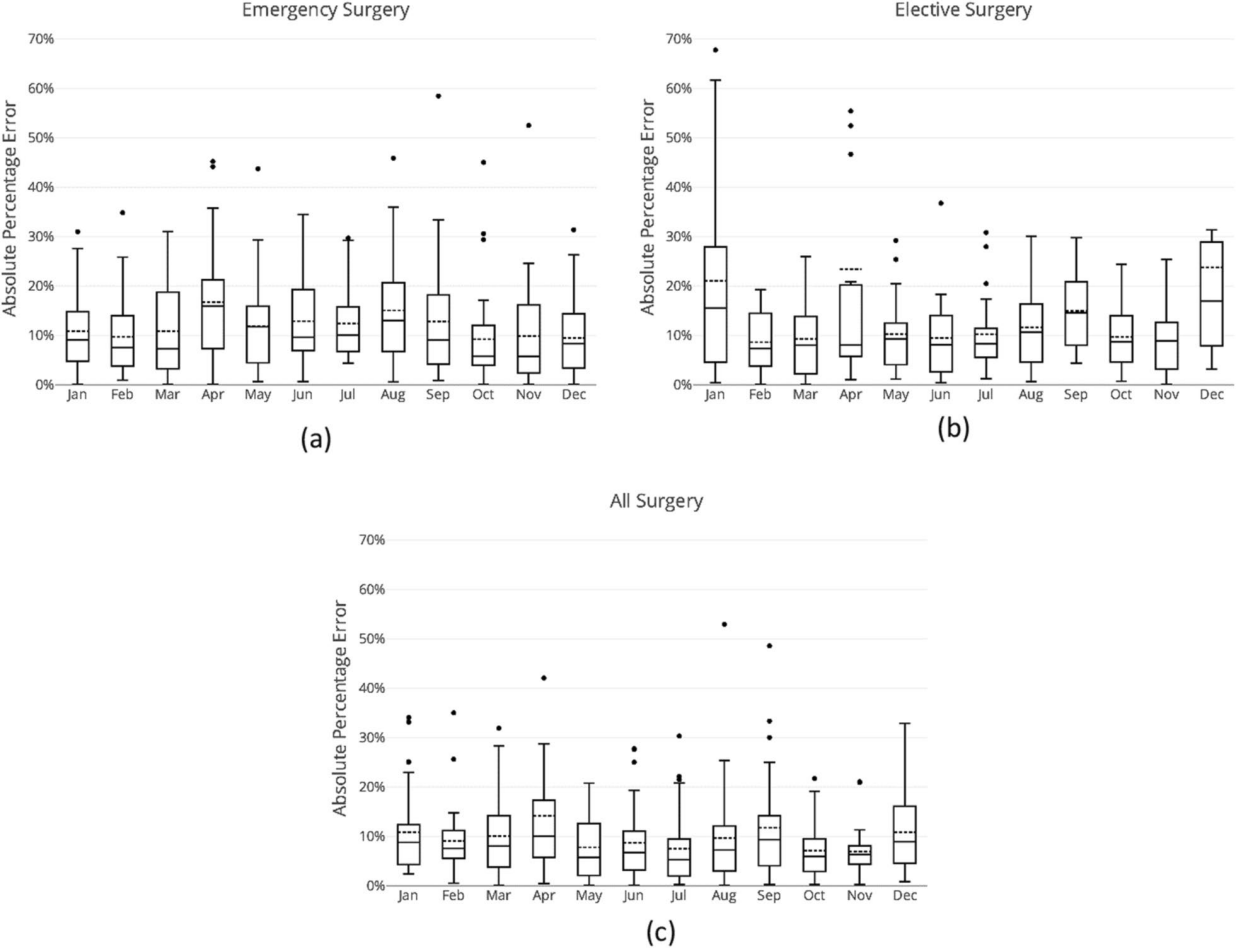
Error per month of year for the Rolling Window approach: **a** emergency surgeries, **b** elective surgeries, and **c** for all surgeries

Figure 9 shows the variation in error by month of year for the Rolling Window approach during the testing period for emergency surgeries (Fig. 9a), for elective surgeries (Fig. 9b), and for all surgeries (Fig. 9c). For the months of April and August, the mean absolute errors of this approach for the prediction of emergency surgeries were 17 and 15% respectively, which were higher than other months (Fig. 9a). This model was able to predict October and December emergency surgery with least error at around 9% mean absolute percentage errors. Fig. 9b shows that the model had highest mean error and widest spread of error when predicting elective surgery demands for the months of January and December with 21 and 24% average error. The average error in April was 23%, however, this was mainly due to a single day (3rd of April 2019) that had a considerably lower number of elective surgeries compared to other working days in the same month (i.e., 24 surgeries on 3rd of April vs. an average of 54 surgeries on other days). Comparing errors of the model in predicting elective versus emergency demands per month of year, it is clear that elective demand was least predictable during summer and holiday seasons (i.e., December and January) while emergency demand was least predictable during autumn and winter.^4^ For overall surgery demand, the model had highest error in April with almost 14% average absolute percentage error and then in September with 12% average error. The most predictable months were October and November with only 7% average absolute error.

### Predicting daily surgery by medical specialty

**Fig. 10:**
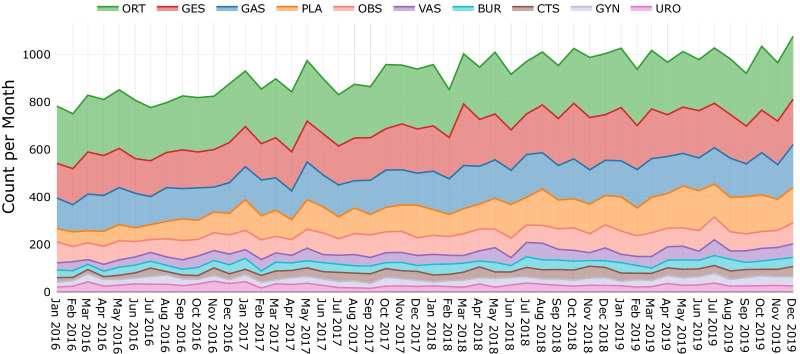
Emergency surgery demand by medical specialty across the study period

Operating theatres can be more efficiently utilised and patients better served if hospitals are informed of the demands of particular surgeries in advance. This section further investigates if historical observations can be used to predict specific surgery demands in terms of clinical specialties. Figure 10 shows emergency surgery demand for the top-10 most frequently performed specialties by month during the study period. Orthopaedic (ORT), General Surgery (GES), and Gastro (GAS) surgeries were the three most prevalent specialties for emergency patients.

**Fig. 11:**
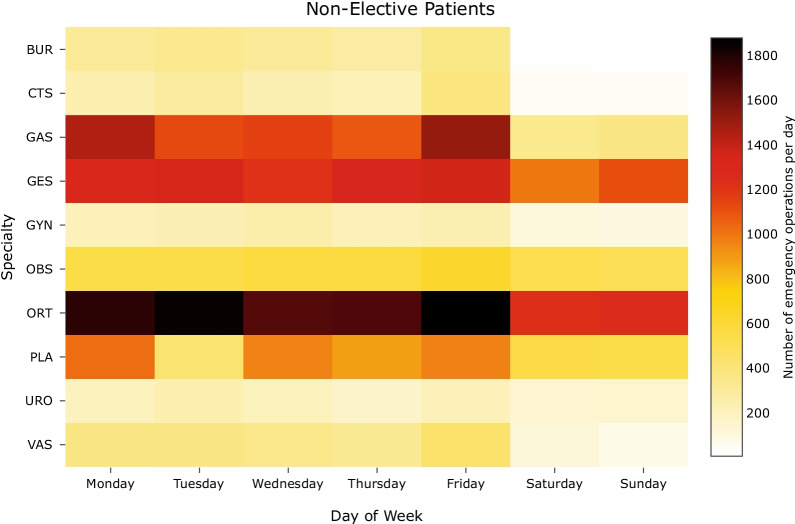
Emergency surgery demands per specialty per day of week

Figure 11 shows the average specialty demands per day of week for the top-10 most frequently performed specialties. It can be observed that the prevalence of some specialties is higher on certain days of the week: GAS surgeries were more frequent on Mondays and Fridays, ORT on Tuesdays and Fridays, and PLA on Mondays, Wednesdays, and Fridays. On average, there were almost similar numbers of GES surgeries throughout the working days in a week. During the weekends, ORT and GES were the two most common specialties while some specialties were only sporadically performed (e.g., BUR, CTS, and GYN).

In order to report MAPE by medical specialty for daily demand prediction, weekends and public holidays were removed from the testing period in addition to days with no surgery for a given specialty. Table 2 shows the results of the Rolling Window approach using different lengths of training data to predict daily demand for specialty-level surgeries. It can be observed that predicting specialty demands on a daily basis was relatively harder for the model than predicting emergency or overall daily demand. The model was able to predict ORT daily emergency demand with almost 80% accuracy using all available training data (i.e., 19.93% MAPE in Table 2). Other emergency specialties were predicted with more than 30% MAPE. Predicting elective specialty demands was generally more difficult than the emergency specialties (over 35% MAPE for the majority of specialties in Table 2). Given that scheduling elective cases is highly dependent on the timetable of surgeons, it is more challenging to identify historical patterns. For example, plastic surgeons at a given hospital may perform operations on a particular day of week in one period of time, which may then shuffle to another day of week. In addition, a number of specialties, such as ORT, are less commonly performed as elective surgeries (e.g., ORT has a daily average of 1 elective case versus 8 emergency cases). Predictive accuracy of overall daily demand for the three most frequently performed specialties, that is, ORT, GES, and GAS, was 20.44, 27.03, and 16.74% MAPE respectively using all available training data. The overall daily caseloads for other medical specialties were predicted with more than 29% MAPE.

**Table 2:** Daily specialty-level surgery demands prediction results (MAPE)

Specialty	Emergency surgery			Elective surgery			Overall surgery		
	1-year %	2-year %	3-year %	1-year %	2-year %	3-year %	1-year %	2-year %	3-year %
ORT	21.25	20.36	19.93	75.05	71.33	70.16	22.65	21.04	20.44
GES	41.38	36.53	34.08	47.37	45.31	47.06	29.88	27.28	27.03
GAS	40.78	37.56	36.32	36.72	33.29	32.59	17.16	16.03	16.74
PLA	40.07	38.59	37.58	47.90	42.73	43.67	29.59	28.29	29.01
OBS	62.21	55.24	52.54	42.56	42.06	39.74	45.33	42.01	39.70
VAS	51.41	45.48	44.17	51.01	48.88	47.43	45.62	42.67	41.52
BUR	45.42	39.97	38.24	51.52	44.51	41.14	44.52	43.87	43.94
URO	43.29	37.69	34.81	54.73	48.24	43.90	53.68	46.95	44.17
CTS	48.15	44.90	41.21	48.35	44.26	42.94	36.15	37.31	35.46
GYN	48.29	43.19	39.31	53.12	45.85	41.20	52.97	48.64	50.43

Although the daily demands of total emergency and overall surgery at a hospital-level could be predicted with approximately 90% accuracy, it is apparent that daily caseloads in smaller subgroups of medical specialties are less predictable. As a result, a practical consideration for generating predictions by medical speciality is providing predictions at a weekly level rather than daily estimates.

## Discussion

### Comparison with state-of-the-art

As mentioned in the Background section, there are few studies that focus on predicting daily surgical caseloads [4, 5]. We found the experimental setting in Zinouri et al. [5] the closest to our study. They report predictive performance of a seasonal ARIMA (SARIMA) of 7% MAPE for a one week ahead forecast horizon and 15.8% MAPE for a 6-month forecast horizon when predicting daily surgical caseload (including elective and non-elective surgeries) during working days (i.e., excluding weekends and public holidays). In contrast, our approach when using the Rolling Window model achieved a MAPE of 9.52% when predicting daily demand across a one year ahead forecast horizon (including weekends and public holidays) using one year of historical observations. Many papers in the forecasting literature compare the fit of a model to the data and do not “hold out” a separate dataset to compare against. Such fitting does not necessarily imply good forecasting, as fitting a high-order polynomial can usually obtain a high level of fit. Overfitting a model is not desirable as it is equivalent to including randomness. In order to overcome this problem, it is better to measure true out-of-sample forecast accuracy. All analyses performed on data in this study were based on training datasets which were compared against a separate held-out evaluation dataset that spanned one year (365 days) which was important in order to assess forecasts over summer and winter months and avoid bias of evaluation at one particular time only.

### Translational outcome

The theatre arrival models developed in this study enables the prediction of theatre demand on a daily basis which can support decision making for improved theatre access for patients. The average compute time of the Rolling Window model was calculated as 6.25 seconds, from the time of training (collecting historical information of 1 to 3 years and weekly window size of 2) to the end of predicting caseloads for one year ahead. The evaluation of the predictive performance of models in this study used a year-ahead forecast horizon to ensure the assessment was not biased for a particular time of year. However, forecasts can be generated for any timeframe: short term use over the weeks ahead is useful for day-to-day theatre management, and long range forecasts can assist with staff recruitment, and inform strategic planning to cater to growth of specific surgery specialties. The models use the most recent data available, and thus any long-range forecasts will be updated with refreshed predictions as new observations become available. The next step is to embed theatre demand models into the hospital’s workflow, allowing operational managers to use the predictive outputs to make theatre workflow more efficient. This will enable hospitals to optimise this high cost resource to achieve consistency and deliver improvements in surgery scheduling, increase theatre utilisation, and reduce cancellations and schedule changes. This can support hospitals in providing services as efficiently and effectively as possible, to obtain the best health outcomes.

### Limitations

**Fig. 12:**
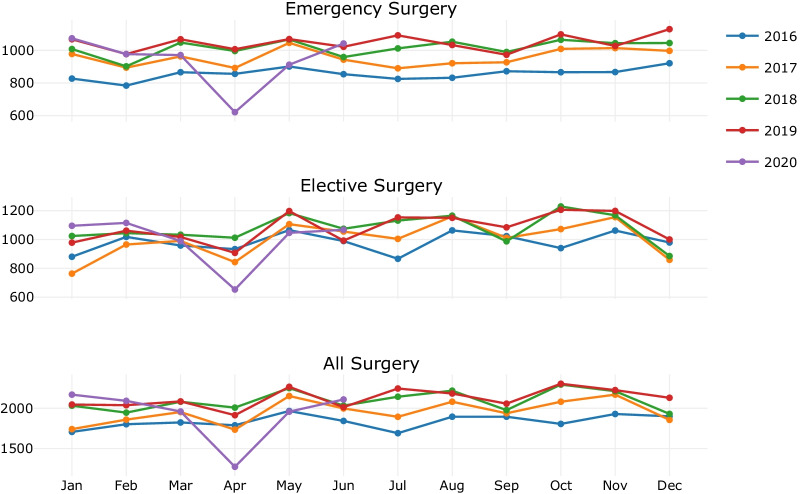
Historical monthly demand–effect of the first wave of the COVID-19 pandemic

The study is based on one hospital in a particular demographic area which may follow a distinct pattern of surgical demand and elective case-booking techniques. A general limitation of predictive models that are based on the historical patterns from a static data extract (e.g., statistical model such as ARIMA or ML approaches) is that they do not consider real-time effects. For example, there was a lockdown period of almost one month in early 2020 due to the COVID-19 pandemic, which started in late March and continued during April 2020. As shown in Fig. 12, surgery demand significantly reduced during the month of April in 2020 as a result of a metropolitan lockdown and restrictions for performing elective surgeries (note that the data in this study contains records until 30 June 2020).

**Fig. 13:**
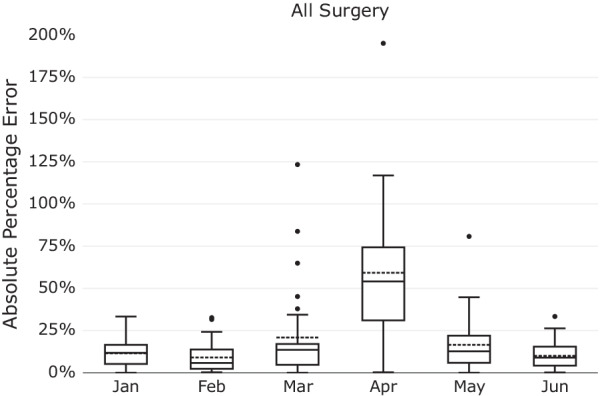
The Rolling Window’s errors in the first 6 months of 2020–COVID-19 effect

Figure 13 shows the result of our demand forecasting model in the first 6 months of 2020. The errors of the model increased during March and April and then again reduced in May and June. MAPE values for these 6 months are as follows (in order): 12%, 9%, 21%, 59%, 17%, 10%. As a decision support tool, such a predictive model will be used by the hospital staff in the context of workflow planning conditions. While such a model may require considerable modification during a pandemic, this limitation does not detract from the usefulness of this model given such temporary unprecedented circumstances [35].

## Conclusion

A model was developed and validated to predict daily arrivals to operating theatres. Unlike approaches such as ARIMA and its extensions that work well for short-term forecasting, the developed model can anticipate long-term demand up to one year ahead with consistent predictive performance throughout this horizon. Further investigation revealed that forecast accuracy depends on the patient cohort to be predicted with variation observed across different medical specialties. When predicting all patient arrivals to theatre on a daily basis, forecast accuracy is 90% (10% error).

This study aimed towards providing a tool for reliably forecasting daily surgery caseload while at the same time addressing a gap in the literature for more detailed analysis of potential approaches for this task in various level of granularity and for short- and long-term forecast horizons. While such a tool can provide insights for staff and resource planning, more translational study is warranted to implement and measure the impact of its outputs on the workflow of operating theatre management team and the magnitude of its benefits for optimising operating theatre efficiency.

## Supplementary Information

**Additional file 1**.** Appendix A**. Regression Model Hyperparameters Tuning.** Appendix B**. Extended Results.

## Data Availability

Data analyzed in this study is unable to be shared due to legislative and review committee requirements. The original data are available from Western Australia’s Department of Health subject to appropriate governance and ethical approvals. Data can be requested from Health Support Services, within the Government of Western Australia (SHaRESupport@health.wa.gov.au, www.hss.health.wa.gov.au).

## Footnotes

1 The study was approved by the FSH QI Medical Anaesthesia & Pain Medicine Committee (Quality activity 29,238) and CSIRO Health and Medical Human Research Ethics Committee (2019_024_LR).

2 The solid lines inside the box plots refer to median and the dotted lines refer to mean.

3 Any inquiry regarding the technical details of the specific models can be made by relevant parties to the corresponding author.

4 Note that, the definition of seasons is based on a southern hemisphere study location.
