# Supplementary material for: Daily surgery caseload prediction: towards improving operating theatre efficiency
Source: BMC Med Inform Decis Mak. 2022 Jun 7;22:151. doi: 10.1186/s12911-022-01893-8 (PMC9172609; doi:10.1186/s12911-022-01893-8)
Supplement: Supplementary file 1 — Additional file 1. Appendix A. Regression Model Hyperparameters Tuning. Appendix B. Extended Results. [file 12911_2022_1893_MOESM1_ESM.docx]

**Daily Surgery Caseload Prediction: Towards Improving Operating Theatre Efficiency (Supplementary Materials)**

**Appendix A: Regression Model Hyperparameters Tuning**

For tuning the hyperparameters, the whole training set was employed to form a tuning development set using data in 2016 and 2017 period for training and the 2018 calendar year for validating/testing. This is due to the fact that, as discussed in the manuscript, a temporal split of the data is more suitable for the prediction task in this study than a cross validation strategy. Note that, the held-out test partition, as described in the manuscript, was not used in the hyperparameter tuning to avoid potential over-fitting. Tuning was done over “overall” operation types and “all” specialties using a Grid Search technique and the mean absolute error as the selection criteria. Table A.1 shows the search space for each parameter and the best selected value in boldface. The default values in these models are as in SciKitLearn library version 0.24.1 and StatModels library version 0.12.2.

Table A.1: Hyperparameters and their values

| **ML Model** | **Parameters and Search Space for Grid Search (best values in boldface)** |
| --- | --- |
| Regression | Family=[**Linear**, Poisson, NegativeBinomial] |
| Decision Tree | criterion= ["squared_error", "friedman_mse", "absolute_error", "**poisson**"]  max_depth= [ **None (default)**, 5,15,25]  min_samples_split= [None (default), 2, **50**, 500] |
| Random Forest | n_estimators= [100, 150, **200**],  max_depth= [ None, 5,15,**25**],  min_samples_split= [2, **50**, 100],  max_features= ["**auto**", "sqrt", "log2"], |
| SVR | Kernel=[**linear**, rbf, sigmoid, poly], used ScikitLearn’s StandardScaler,  Gamma= ["**scale**", "auto"],  C=[**1.0**, 1e-3, 1e-2, 1e-1, 10, 100],  Shrinking= [True, **False**],  Degree= [None, 1, 5] # for poly kernel only |
| Bagging Regressor | n_estimators= [**5**, 10, 20],  max_samples= [0.5, **1.0**],  max_features= [0.5, **1.0**], |
| Gradient Boosting Regressor | loss= ['squared_error', 'absolute_error', 'huber', **'quantile'**],  learning_rate= [1e-3, 1e-2, **0.1**, 1.0],  n_estimators= [50, 100, **200**], |
| Ensemble Regressor | base_estimator= BaggingRegressor, GradientBoostingRegressor, RandomForestRegressor, parameters as in individual models above |
| XGBoost Regressor | tree_method= ['gpu_hist', 'approx', **'hist'**],  learning_rate= [1e-3, 1e-2, **0.1**, 1.0],  n_estimators= [**50**, 100, 200],  max_depth= [**5**,10,15,25], |

**Appendix B. Extended Results**

Table B.1: Rolling window on historic observations - Uniform weight averaging - Full results

| Cohort | Yearly Frame ($\tau$) | Weekly Window ($\theta$) | MAE | MSE | RMSE | MAPE |
| --- | --- | --- | --- | --- | --- | --- |
| All Patients | 1 | 1 | 6.5404 | 89.8768 | 9.4803 | 0.1028 |
| All Patients | 2 | 1 | 6.3254 | 69.3595 | 8.3282 | 0.1011 |
| All Patients | 3 | 1 | 7.3338 | 86.8705 | 9.3204 | 0.1142 |
| All Patients | 1 | 2 | 5.9984 | 68.4957 | 8.2762 | 0.0952 |
| All Patients | 2 | 2 | 6.1688 | 66.0739 | 8.1286 | 0.0983 |
| All Patients | 3 | 2 | 7.292 | 84.9098 | 9.2146 | 0.1131 |
| All Patients | 1 | 3 | 6.0482 | 66.6659 | 8.1649 | 0.0952 |
| All Patients | 2 | 3 | 6.2389 | 66.6787 | 8.1657 | 0.0984 |
| All Patients | 3 | 3 | 7.3461 | 86.6013 | 9.306 | 0.1135 |
| Emergency Patients | 1 | 1 | 4.4183 | 35.6335 | 5.9694 | 0.1292 |
| Emergency Patients | 2 | 1 | 4.3132 | 29.7968 | 5.4586 | 0.1262 |
| Emergency Patients | 3 | 1 | 4.8685 | 36.3844 | 6.0319 | 0.1399 |
| Emergency Patients | 1 | 2 | 4.002 | 27.2016 | 5.2155 | 0.1179 |
| Emergency Patients | 2 | 2 | 4.1395 | 28.889 | 5.3748 | 0.1205 |
| Emergency Patients | 3 | 2 | 4.7712 | 35.6325 | 5.9693 | 0.1366 |
| Emergency Patients | 1 | 3 | 3.9831 | 26.5346 | 5.1512 | 0.117 |
| Emergency Patients | 2 | 3 | 4.1102 | 28.5837 | 5.3464 | 0.1195 |
| Emergency Patients | 3 | 3 | 4.7424 | 35.5496 | 5.9624 | 0.1357 |
| Elective Patients | 1 | 1 | 6.8078 | 79.1883 | 8.8988 | 0.137 |
| Elective Patients | 2 | 1 | 6.4728 | 69.1558 | 8.316 | 0.1243 |
| Elective Patients | 3 | 1 | 6.6957 | 72.4475 | 8.5116 | 0.1274 |
| Elective Patients | 1 | 2 | 6.5696 | 74.6183 | 8.6382 | 0.1326 |
| Elective Patients | 2 | 2 | 6.4327 | 69.2744 | 8.3231 | 0.1261 |
| Elective Patients | 3 | 2 | 6.7163 | 73.0108 | 8.5446 | 0.1297 |
| Elective Patients | 1 | 3 | 6.7164 | 76.9061 | 8.7696 | 0.1365 |
| Elective Patients | 2 | 3 | 6.5406 | 72.8474 | 8.5351 | 0.1294 |
| Elective Patients | 3 | 3 | 6.912 | 77.8724 | 8.8245 | 0.1345 |

Table B.2: Regression Errors on All Patients

| Approach | Yearly Frame | MAE | MSE | RMSE | MAPE |
| --- | --- | --- | --- | --- | --- |
| Random Forest Regressor | 2 | 5.6454 | 67.3118 | 8.2044 | 0.0971 |
| Random Forest Regressor | 3 | 5.7302 | 68.9683 | 8.3047 | 0.0976 |
| Ensemble Regressor | 3 | 5.8039 | 73.3833 | 8.5664 | 0.0982 |
| Random Forest Regressor | 1 | 5.7762 | 69.8813 | 8.3595 | 0.0983 |
| Ensemble Regressor | 2 | 5.9435 | 72.8312 | 8.5341 | 0.0997 |
| Bagging Regressor | 3 | 5.9723 | 77.928 | 8.8277 | 0.1026 |
| Ensemble Regressor | 1 | 6.1931 | 80.8057 | 8.9892 | 0.1033 |
| Bagging Regressor | 2 | 6.214 | 82.3208 | 9.0731 | 0.1042 |
| Gradient Boosting Regressor | 3 | 6.4384 | 85.296 | 9.2356 | 0.1061 |
| Bagging Regressor | 1 | 6.3748 | 82.6744 | 9.0925 | 0.1062 |
| Gradient Boosting Regressor | 1 | 6.4459 | 88.0465 | 9.3833 | 0.1074 |
| Gradient Boosting Regressor | 2 | 6.9473 | 93.9062 | 9.6905 | 0.1121 |
| XGB Regressor | 3 | 6.1307 | 79.6102 | 8.9225 | 0.1122 |
| XGB Regressor | 2 | 7.1427 | 99.1334 | 9.9566 | 0.1164 |
| DecisionTreeRegressor | 3 | 7.463 | 107.611 | 10.3736 | 0.1214 |
| Regression | 3 | 7.8297 | 118.5099 | 10.8862 | 0.1285 |
| Regression | 2 | 8.3719 | 151.7231 | 12.3176 | 0.1349 |
| Regression (Poisson) | 3 | 8.3786 | 132.3828 | 11.5058 | 0.1377 |
| Regression (Negative Binomial) | 3 | 8.4483 | 135.429 | 11.6374 | 0.1386 |
| DecisionTreeRegressor | 2 | 7.6356 | 106.6986 | 10.3295 | 0.1397 |
| Regression (Poisson) | 2 | 8.6765 | 153.7998 | 12.4016 | 0.1399 |
| Regression (Negative Binomial) | 2 | 8.7393 | 155.8467 | 12.4839 | 0.1408 |
| XGB Regressor | 1 | 10.4679 | 210.1543 | 14.4967 | 0.1506 |
| DecisionTreeRegressor | 1 | 10.9315 | 251.337 | 15.8536 | 0.1599 |
| SRV (Linear) | 1 | 19.0963 | 603.2012 | 24.5602 | 0.3581 |
| SRV (Poly) | 3 | 20.694 | 757.2901 | 27.5189 | 0.3643 |
| SRV (Linear) | 3 | 18.9971 | 580.6465 | 24.0966 | 0.3696 |
| SRV (Linear) | 2 | 19.0314 | 576.6271 | 24.0131 | 0.3793 |
| SRV (RBF) | 3 | 22.6498 | 638.913 | 25.2767 | 0.4815 |
| SRV (Poly) | 2 | 26.413 | 1113.331 | 33.3666 | 0.4884 |
| SRV (Sigmoid) | 2 | 21.2231 | 635.9654 | 25.2184 | 0.5005 |
| SRV (Sigmoid) | 3 | 21.6621 | 669.7117 | 25.8788 | 0.5149 |
| SRV (RBF) | 2 | 26.4752 | 803.25 | 28.3417 | 0.574 |
| SRV (Sigmoid) | 1 | 23.5037 | 746.663 | 27.3251 | 0.6037 |
| SRV (RBF) | 1 | 28.2825 | 1009.7322 | 31.7763 | 0.7468 |
| SRV (Poly) | 1 | 43.51 | 3549.6808 | 59.5792 | 0.9015 |

Table B.3: Regression Errors on Emergency Patients

| Approach | Yearly Frame | MAE | MSE | RMSE | MAPE |
| --- | --- | --- | --- | --- | --- |
| Random Forest Regressor | 2 | 3.6413 | 23.4811 | 4.8457 | 0.1103 |
| Random Forest Regressor | 3 | 3.6594 | 23.8923 | 4.888 | 0.1108 |
| Random Forest Regressor | 1 | 3.6416 | 23.1994 | 4.8166 | 0.1114 |
| Gradient Boosting Regressor | 3 | 3.6993 | 23.5842 | 4.8564 | 0.1127 |
| Ensemble Regressor | 2 | 3.7539 | 24.5867 | 4.9585 | 0.1146 |
| Ensemble Regressor | 3 | 3.8304 | 25.2002 | 5.02 | 0.1161 |
| Gradient Boosting Regressor | 2 | 3.7989 | 24.4703 | 4.9468 | 0.1169 |
| Bagging Regressor | 2 | 3.9351 | 27.2871 | 5.2237 | 0.1197 |
| Ensemble Regressor | 1 | 4.0029 | 27.0148 | 5.1976 | 0.1236 |
| Bagging Regressor | 3 | 4.1589 | 29.3728 | 5.4197 | 0.125 |
| Bagging Regressor | 1 | 4.2003 | 28.9861 | 5.3839 | 0.1286 |
| Gradient Boosting Regressor | 1 | 4.2586 | 30.9662 | 5.5647 | 0.1325 |
| XGB Regressor | 1 | 4.416 | 31.211 | 5.5867 | 0.136 |
| XGB Regressor | 2 | 4.4038 | 31.2339 | 5.5887 | 0.1365 |
| Regression | 2 | 4.4427 | 32.9583 | 5.7409 | 0.1371 |
| Regression | 3 | 4.4624 | 33.4036 | 5.7796 | 0.1379 |
| Regression (Negative Binomial) | 2 | 4.5459 | 34.2574 | 5.853 | 0.1413 |
| Regression (Poisson) | 2 | 4.5574 | 34.4156 | 5.8665 | 0.1417 |
| Regression (Poisson) | 3 | 4.735 | 36.8745 | 6.0724 | 0.1471 |
| Regression (Negative Binomial) | 3 | 4.8006 | 38.0262 | 6.1665 | 0.1488 |
| XGB Regressor | 3 | 4.7466 | 36.7763 | 6.0643 | 0.1489 |
| DecisionTreeRegressor | 1 | 5.0822 | 40.5178 | 6.3654 | 0.1569 |
| SRV (Linear) | 2 | 5.4507 | 54.223 | 7.3636 | 0.165 |
| SRV (Linear) | 3 | 5.6019 | 53.5071 | 7.3149 | 0.175 |
| SRV (RBF) | 3 | 6.2266 | 62.3766 | 7.8979 | 0.1786 |
| DecisionTreeRegressor | 2 | 6.1068 | 59.1863 | 7.6933 | 0.1797 |
| DecisionTreeRegressor | 3 | 6.1288 | 59.411 | 7.7079 | 0.183 |
| SRV (Linear) | 1 | 5.9321 | 55.3046 | 7.4367 | 0.1913 |
| SRV (RBF) | 2 | 6.6092 | 66.1019 | 8.1303 | 0.1937 |
| SRV (Sigmoid) | 1 | 6.5237 | 64.2481 | 8.0155 | 0.2049 |
| SRV (RBF) | 1 | 7.48 | 77.9381 | 8.8283 | 0.2235 |
| SRV (Poly) | 3 | 7.4327 | 75.5946 | 8.6945 | 0.2363 |
| SRV (Poly) | 2 | 9.0632 | 130.8814 | 11.4403 | 0.2667 |
| SRV (Poly) | 1 | 15.4217 | 534.3228 | 23.1154 | 0.4412 |
| SRV (Sigmoid) | 3 | 15.8923 | 404.363 | 20.1088 | 0.5095 |
| SRV (Sigmoid) | 2 | 15.9579 | 449.5088 | 21.2016 | 0.5191 |

Table B.4: Regression Errors on Elective Patients

| Approach | Yearly Frame | MAE | MSE | RMSE | MAPE |
| --- | --- | --- | --- | --- | --- |
| Random Forest Regressor | 2 | 5.8046 | 57.5642 | 7.5871 | 0.1143 |
| Gradient Boosting Regressor | 1 | 6.1623 | 60.7821 | 7.7963 | 0.1163 |
| Bagging Regressor | 2 | 5.9199 | 58.3285 | 7.6373 | 0.119 |
| Random Forest Regressor | 3 | 6.6517 | 73.4223 | 8.5687 | 0.125 |
| Ensemble Regressor | 2 | 6.2438 | 63.0661 | 7.9414 | 0.1269 |
| Ensemble Regressor | 1 | 7.2152 | 84.9789 | 9.2184 | 0.1299 |
| Ensemble Regressor | 3 | 7.222 | 85.3829 | 9.2403 | 0.133 |
| SRV (RBF) | 1 | 6.6243 | 78.007 | 8.8322 | 0.136 |
| Bagging Regressor | 1 | 7.6032 | 94.9798 | 9.7458 | 0.1362 |
| SRV (RBF) | 2 | 6.7464 | 79.3431 | 8.9075 | 0.1365 |
| SRV (RBF) | 3 | 6.6811 | 76.9904 | 8.7744 | 0.1378 |
| DecisionTreeRegressor | 2 | 7.2789 | 87.3586 | 9.3466 | 0.1391 |
| Gradient Boosting Regressor | 3 | 7.5846 | 95.2284 | 9.7585 | 0.14 |
| Bagging Regressor | 3 | 7.8618 | 96.7378 | 9.8355 | 0.1425 |
| DecisionTreeRegressor | 1 | 7.6454 | 100.1554 | 10.0078 | 0.1455 |
| DecisionTreeRegressor | 3 | 7.5219 | 90.1833 | 9.4965 | 0.1472 |
| SRV (Linear) | 3 | 6.9755 | 87.5243 | 9.3554 | 0.1519 |
| SRV (Sigmoid) | 1 | 7.104 | 91.1208 | 9.5457 | 0.1519 |
| SRV (Poly) | 3 | 7.4173 | 90.5494 | 9.5157 | 0.1555 |
| SRV (Linear) | 1 | 7.1437 | 91.73 | 9.5776 | 0.1561 |
| SRV (Linear) | 2 | 7.1353 | 91.5523 | 9.5683 | 0.1562 |
| XGB Regressor | 2 | 8.6192 | 119.4497 | 10.9293 | 0.1574 |
| Regression | 3 | 7.7421 | 96.3394 | 9.8153 | 0.1584 |
| Gradient Boosting Regressor | 2 | 8.1148 | 100.1503 | 10.0075 | 0.1652 |
| Regression (Negative Binomial) | 3 | 8.2001 | 107.406 | 10.3637 | 0.1667 |
| Regression (Poisson) | 3 | 8.1896 | 106.5879 | 10.3241 | 0.1668 |
| Regression | 2 | 8.7041 | 121.1744 | 11.0079 | 0.1779 |
| Random Forest Regressor | 1 | 10.0767 | 142.4406 | 11.9348 | 0.1785 |
| Regression (Poisson) | 2 | 9.283 | 140.2527 | 11.8428 | 0.189 |
| SRV (Sigmoid) | 2 | 8.916 | 145.6495 | 12.0685 | 0.1894 |
| Regression (Negative Binomial) | 2 | 9.4011 | 144.3694 | 12.0154 | 0.1912 |
| SRV (Poly) | 1 | 9.2065 | 136.4604 | 11.6816 | 0.1923 |
| SRV (Sigmoid) | 3 | 10.1749 | 180.6413 | 13.4403 | 0.2102 |
| SRV (Poly) | 2 | 10.2728 | 157.2043 | 12.5381 | 0.2131 |
| XGB Regressor | 1 | 20.8106 | 501.2382 | 22.3883 | 0.3718 |
| XGB Regressor | 3 | 22.6374 | 611.2111 | 24.7227 | 0.4092 |
